# Deep learning-based enhancement of fluorescence labeling for accurate cell lineage tracing during embryogenesis

**DOI:** 10.1093/bioinformatics/btae626

**Published:** 2024-10-17

**Authors:** Zelin Li, Dongying Xie, Yiming Ma, Cunmin Zhao, Sicheng You, Hong Yan, Zhongying Zhao

**Affiliations:** Department of Electrical Engineering, City University of Hong Kong, Hong Kong 999077, China; Centre for Intelligent Multidimensional Data Analysis, Hong Kong Science Park, Hong Kong 999077, China; Department of Biology, Hong Kong Baptist University, Hong Kong 999077, China; Department of Biology, Hong Kong Baptist University, Hong Kong 999077, China; Department of Biology, Hong Kong Baptist University, Hong Kong 999077, China; Centre for Intelligent Multidimensional Data Analysis, Hong Kong Science Park, Hong Kong 999077, China; Department of Electrical Engineering, City University of Hong Kong, Hong Kong 999077, China; Centre for Intelligent Multidimensional Data Analysis, Hong Kong Science Park, Hong Kong 999077, China; Department of Biology, Hong Kong Baptist University, Hong Kong 999077, China

## Abstract

**Motivation:**

Automated cell lineage tracing throughout embryogenesis plays a key role in the study of regulatory control of cell fate differentiation, morphogenesis and organogenesis in the development of animals, including nematode *Caenorhabditis elegans*. However, automated cell lineage tracing suffers from an exponential increase in errors at late embryo because of the dense distribution of cells, relatively low signal-to-noise ratio (SNR) and imbalanced intensity profiles of fluorescence images, which demands a huge amount of human effort to manually correct the errors. The existing image enhancement methods are not sensitive enough to deal with the challenges posed by the crowdedness and low signal-to-noise ratio. An alternative method is urgently needed to assist the existing detection methods in improving their detection and tracing accuracy, thereby reducing the huge burden for manual curation.

**Results:**

We developed a new method, termed as *DELICATE*, that dramatically improves the accuracy of automated cell lineage tracing especially during the stage post 350 cells of *C. elegans* embryo. *DELICATE* works by increasing the local SNR and improving the evenness of nuclei fluorescence intensity across cells especially in the late embryos. The method both dramatically reduces the segmentation errors by StarryNite and the time required for manually correcting tracing errors up to 550-cell stage, allowing the generation of accurate cell lineage at large-scale with a user-friendly software/interface.

**Availability and implementation:**

All images and data are available at https://doi.org/10.6084/m9.figshare.26778475.v1. The code and user-friendly software are available at https://github.com/plcx/NucApp-develop.

## 1 Introduction

Automated cell lineage tracing using fluorescence markers is a revolutionary technique in developmental biology (Fire 1994, Bao *et al.* 2006, Kretzschmar and Watt 2012, Mcdole *et al.* 2018, Malin-Mayor *et al.* 2022), providing unprecedented insights into the intricate regulation of cell division and differentiation, tissue formation and organogenesis within a developing embryo. These methods employ fluorescent proteins to tag cells, thereby enabling researchers to monitor the progeny of individual cells when they undergo rapid division, allowing precise tracing of cell division over cell cycle, which results in a complete picture of cell division history, or so-called “cell lineage”. The results provide an in-depth view of cellular behaviors at exceptionally high temporal resolution, including cell division and cell cycle length, cell migration, cell fate differentiation, cell-cell contact, cellular signaling, cellular shape dynamics, and lineal expression pattern of gene in the developing organism (Li *et al.* 2019, Cao *et al.* 2020, Ma *et al.* 2021, Thiels *et al.* 2021, Guan *et al.* 2023).

*Caenorhabditis elegans* embryo is a model of choice for automated cell lineage analysis due to its transparent body and invariant cell lineage throughout development on top of the availability of abundant molecular and genetic tools (Brenner 1974, Sulston *et al.* 1983, Fire *et al.* 1998, Murray *et al.* 2008, Frøkjær-Jensen *et al.* 2014). Its cell lineage tracing has been greatly facilitated using ubiquitously labeled nuclei with fluorescent proteins such as green fluorescent protein (GFP). The time-lapse 3D fluorescence image stacks were used as an input for customized image processing and visualization programs, StarryNite and AceTree, respectively (Boyle *et al.* 2006, Murray *et al.* 2006, Santella *et al.* 2010, Santella *et al.* 2014, Katzman *et al.* 2018). These tools allows for automatically tracing and visualizing the cell lineage from the single-celled zygote up to approximately 550-cell stage of a developing embryo when most cells complete their last round of division and are committed to their terminal fate during embryogenesis (Sulston *et al.* 1983).

A key challenge in automated cell lineage tracing of *C. elegans* is the accurate detection and segmentation of densely packed, small embryonic cells, particularly during later developmental stages. As cells proliferate, their closely positioned nuclei make it difficult to distinguish neighboring individuals based on fluorescence alone. This issue is exacerbated by the small size and rapid division of *C. elegans* cells. In addition, photobleaching and phototoxicity effects during the prolonged imaging can reduce fluorescence intensity and potentially alter embryo development, resulting in poor image quality. The heterogeneity in nuclear size and marker intensity across cell types and lineages further complicates accurate nucleus detection and segmentation. For example, the commonly used histone-fluorescent protein fusion marker (his-72::GFP/mCherry) exhibits significantly lower expression in the D, E, and germline progenitor (P4, Z2, Z3) sublineages, leading to false-negative calls (Bao *et al.* 2006). Even using alternative lineaging strains only partially mitigates this problem. The relatively large nuclei of the E lineage also frequently result in over-segmentation. Consequentially, the output of StarryNite suffers from numerous errors, ranging from incorrect call of a cell division to missing a cell or division, especially in a late embryo (Aydin *et al.* 2010, Santella *et al.* 2010, Santella *et al.* 2014, Katzman *et al.* 2018). Therefore, substantial manual correction by experts is required to address the various errors, making large-scale cell lineage analyses tedious and labor-intensive.

A previous attempt was made to automatically correct a subset of the errors made by StarryNite using machine learning (Aydin *et al.* 2010). However, the method only dealt with a single class of error, i.e. cell movement was wrongly annotated as divisions, which were produced before 200-cell stage. Substantial amount of time for manual correction is still required for various other types of errors, especially those produced beyond 200-cell stage. A more robust method is needed to reduce the errors not only produced by cell movement but also produced by other factors detailed below.

To close this gap, we developed a novel pipeline, termed deep learning-based cell segmentation enhancement technology (*DELICATE*), to improve the nuclei fluorescence images. As a light-weight deep learning network and data-driven model, *DELICATE* is customized for *C. elegans* with a relatively small number of time-series imaged embryos (Supplementary Table S1), i.e. six training time-lapse embryos (1270 3D volumes), providing much-improved image quality for accurate cell lineage tracing up to the 550-cell stage. The method enhanced the 2D images via 3D segmentor-DNN, which improves the SNR, fluorescent signal intensity, and the effective information content of the processed images acquired under different imaging conditions, allowing curation of cell lineage up to the 550-cell stage more efficiently.

## 2 Materials and methods

### 2.1 Image acquisition

All embryos used in this study were *C. elegans* transgenic strain RW10029 (Bao *et al.* 2006) with the genotype: *unc-119(ed3)* III; zuIs178 [his-72(1 kb 5' UTR)::his-72::SRPVAT::GFP::his-72 (1KB 3' UTR) + 5.7 kb XbaI—HindIII unc-119(+)]; stIs10024 [pie-1::H2B::GFP::pie-1 3' UTR + unc-119(+)]. Time-lapse 3D images were acquired for a total of 9 embryos (Supplementary Table S1), including 4 imaged under pressure by cover slide while the remaining 5 without pressure. All the compressed images were generated previously (Cao *et al.* 2020). To compare embryos labeled with a different fluorescence marker, we used an embryo of another *C. elegans* transgenic strain RW10425 with the genotype: unc-119(ed3) III; stIs10116 [his-72(promoter)::his-24::mCherry::let-858 3'UTR + unc-119(+)]; stIs37 [pie-1(promoter)::mCherry::H2B::pie-1 3'UTR + unc-119(+)]; stIs10389 [PHA-4::TGF(3E3)::GFP::TY1::3xFLAG], from our previously published data (Ho *et al.* 2015).

To ensure that the embryo is not compressed during imaging, a solution of polylysine (1 *ng/ml*) and Boyd’s buffer was mixed in equal volume ratio. Approximately 7 *µl* of this mixture was carefully pipetted onto a glass slide and spread to form a rectangular pad with roughly 0.7 *cm* × 1 *cm*. This slide was then placed onto a heating block set at 60*°C* to dry the pad. Following the drying process, small dots of Vaseline were deposited just outside each corner of the polylysine-Boyd’s buffer pad to serve as supports for a cover slip.

The embryo dissection followed the standard protocols for dissecting embryos (Murray *et al.* 2006). Next, a tiny drop of Boyd’s buffer was placed onto the prepared pad, where it was observed to spread within the confined area. Embryos were then transferred individually using a capillary mouth needle, ensuring that any excess buffer was expelled from the needle tip prior to transfer. After the embryos were blown out onto the pad, they were allowed to settle on the slide for 3–5*s* before mounting the next embryo. Once all embryos were positioned on the pad, they were left to settle for an additional 20–30*s* before being covered with 12–15 *µl* of Boyd’s buffer. A cover slip was then gently laid over the embryos, resting on the Vaseline dots to avoid direct contact with the buffer. The cover slip was then methodically pressed at each corner until it contacted the buffer, ensuring that the embryos remained within the intended area. To finalize the mounting preparation, the edges of the cover slip were sealed with molten Vaseline, creating an airtight environment to maintain the sample's integrity for imaging.

For acquisition of 3D time-lapse images without pressure, a total of five embryos were used (Supplementary Table S1). The GFP images were acquired using a Leica SP8 confocal microscope under constant temperature of 20°C with a frame size of 712×512 pixels (*x*/*y* resolution: 0.09 *μm*) and a scanning speed of 8000 *Hz* using a water immersion objective len. An excitation laser of 488 *nm* was used for GFP illumination. A total of 92 focal planes were sequentially collected for five embryos without pressure per time point (approximately 1.5 *min*), with a *z* resolution of approximately 0.42 *μm* (Supplementary Table S1). Imaging was conducted continuously over at least 240 time points. The imaging was divided into five time-blocks to accommodate the intensity changes in fluorescence signal during development. Five different blocks with different pinholes and gains were set according to the fluorescence intensity: Block1 (60 time points): pinhole: 2.7, detection gain: 110; Block2 (30 time points): pinhole: 2.4, detection gain: 80; Block3 (40 time points): pinhole: 2.1, detection gain: 70; Block4 (30 time points): pinhole: 1.8, detection gain: 70; Block5 (90 time points): pinhole: 1.6, detection gain: 40. The images were subject to image deconvolution with Huygens (Huygens Essential, Scientific Volume Imaging BV) for initial image processing.

### 2.2 Cell lineage tracing and generation of ground truth by manual annotation

The automated cell lineage tracing results were generated by StarryNite as described (Murray *et al.* 2006) and manually corrected up to the 550-cell stage when C lineage progeny completes all divisions using AceTree (Boyle *et al.* 2006). All the images were applied with the same StarryNite parameters (Supplementary Table S2) except for the “intensitythreshold,” which is [0.004,0.005,0.008,0.001,0.008,0.008] for raw images and [0.02,0.015,0.04,0.039,0.039,0.04] for the enhanced images; The corrected tracing data were saved as CD files and used as the ground truth (GT) for the subsequent training. The cell lineage data for a total of six embryos with or without pressure (Supplementary Table S1) were thoroughly curated from at least 4- to 550-cell stage of embryogenesis, including the *xyz* positions and sizes for all cell nuclei (Supplementary Fig. S1). Based on the curated cell nuclei positions and the corresponding size (radius), a sphere of the corresponding size was rendered to represent cell nuclei and placed to the same locations for all cell nuclei in the 3D GT images. Collectively, there were a total 1270 3D volumetric images (the embryogenesis by 550-cell stage) in the training dataset for the segmentor-DNN training, which was corresponding to 98 360 2D images from the 6 embryos. The training data covered images of a range of quality acquired with or without pressure applied on the mounted embryos, which make the data-driven segmentor-DNN, as well as our enhancement method *DELICATE*, can accommodate various fluorescence images under different imaging conditions. Moreover, we also trained and evaluated our pipeline with five embryos from other groups (Li *et al.* 2019, Ma *et al.* 2021) (Supplementary Table S1).

### 2.3 Implementation: DNN structure, image processing and training

The proposed method and the corresponding software were implemented with TensorFlow, StarDist, and Pyqt5 (Schmidt *et al.* 2018, Weigert *et al.* 2020). All programs were built with Python (v3.8). We used the built-in Res-Net of segmentor-DNN from TensorFlow and StarDist, which provides a stable segmentor-DNN. In addition, we developed a user-friendly interface software for researchers to enhance their own nuclei fluorescence images without programming. The software interface with complicated algorithms and processing steps was described in detail below.

The segmentor-DNN applied U-Net architecture of depth 4 (Ronneberger *et al.* 2015), which allows the learning of significant spatial information in biomedical images. The integrated model contains multiple residual blocks of Res-Net that enable both fusion and communication between early (shallow) and late (deep) layer (Supplementary Fig. S1). The model takes the single 3D volumetric image input as a probability map of one cell object and the radial distance to the cell nuclei boundary in star-convex polygon form. Consequently, the voxels with a high probability (larger than an automatically generated threshold) are recognized as cell nucleus’ boundary, as a polygon with 64 rays. A nonmaximum suppression is finally applied for pruning redundant overlapped nuclei. Following this, multiple cell nuclei regions in the 3D image are uniquely segmented, facilitating the following steps of fluorescence image enhancement and merging.

The inputs for the segmentor-DNN were GFP-labeled nuclei fluorescence images, with the corresponding ground truth (GT) images serving as the learning/fitting targets. During training, the input image $\left(I_{\mathrm{input}}\right)$ underwent random noise addition, was cropped to a volumetric size of (128 ×128 ×128), and flipped to augment the dataset, thereby enhancing the network’s robustness. The Adam optimizer was employed with an initial learning rate of $\left(5\times 10^{-3}\right)$ and a weight decay rate of $\left(1\times 10^{-5}\right)$, complemented by AMSGrad for gradient descent optimization, to update the network. The model underwent training over 400 epochs with a batch size of 8, utilizing an NVIDIA 2080 Ti GPU. The loss function is defined as ${L_{\mathrm{nuc}}\left(P,\;G\right)=L}_{\mathrm{obj}}+\eta L_{\mathrm{dist}}$ (Weigert *et al.* 2020). The segmentor-DNN automatically choose the final parameters with smallest validation loss during training to reduce the overfitting risks.

*DELICATE* aims to improve the accuracy of nuclei detection and cell lineage construction by StarryNite, while keeping the necessary fluorescence signals and most information of cell nuclei to facilitate human intervention. Thus, we merge the segmented pseudo-nuclei $I_{\mathrm{pseudo}}$ (prediction in Supplementary Fig. S1) into raw 2D fluorescence images $I_{\mathrm{raw}}$ to enhance the ambiguous and unseen areas with $\Phi \left(I_{\mathrm{raw}},I_{\mathrm{pseudo}}\right)$ for every $v_{i}$ in $I_{\mathrm{raw}}$, i.e. defined as:

ΦIraw,Ipseudo|vi=vi in Iraw, vi of Ipseudo is 0max(Iraw), else

The enhanced images contained both fluorescence signals and pseudo-nuclei information. The input (from deconvolution software) and output (compatible with StarryNite) images were both 2D images in TIF format, which is compatible with StarryNite (Murray *et al.* 2006, Santella *et al.* 2010, 2014). The interface runs the enhancement for one embryo once, taking ∼0.5 h to finish the whole automated process.

### 2.4 Quantification of mean square error (MSE), signal-to-noise ratio (SNR), precision, accuracy and recall

MSE, based on pixels, and SNR, based on the whole image, are formulated respectively as:

$$\begin{array}{c} MSE=\frac{1}{mn}\sum_{i=1}^{m}\sum_{j=1}^{n}{\left(I_{\mathrm{gt}}\left(i,\;j\right),\;I\left(i,\;j\right)\right)}^{2}\\ SNR=\frac{P_{\mathrm{signal}}}{P_{\mathrm{noise}}} \end{array}$$

The $P_{\mathrm{signal}}$ and $P_{\mathrm{noise}}$ are the power of signals and noises in the images, the total intensity of the corresponding pixels. In this study, the signals are the intensity of voxels within all cell nuclei regions (edited manually) marked in the GT images, while the noise is the outside the regions. *MSE* and *SNR* represent the “useful” signals level for cellular tracing in images.

Precision, recall, and accuracy at every single time point are formulated as:

$$\begin{array}{c} P\mathrm{recision}=\frac{\mathrm{True}\;\mathrm{Positives}}{\mathrm{True}\;\mathrm{Positives}\;+\;\mathrm{False}\;\mathrm{Positives}},\;\\ \begin{array}{c} Rec\mathrm{all}=\frac{\mathrm{True}\;\mathrm{Positives}}{\mathrm{True}\;\mathrm{Positives}\;+\;\mathrm{False}\;\mathrm{Negatives}},\;\\ A\mathrm{ccuracy}=\frac{\mathrm{True}\;\mathrm{Positives}}{\mathrm{True}\;\mathrm{Positives}\;+\;\mathrm{False}\;\mathrm{Positives}\;+\;\mathrm{False}\;\mathrm{Negatives}}. \end{array} \end{array}$$

To compared cellular lineage tracing ground truth, True Positives and False Positives are the correctly or incorrectly detected cells in a single time point, respectively. False Negatives are the missing detected cells that should be traced in the ground truth lineage tree and no True Negatives exists (0 for lineaging tracing). Thus, higher values metrics represent better lineage tracing performances.

## 3 Results

### 3.1 Characterization of errors made by StarryNite

**Figure 1.**
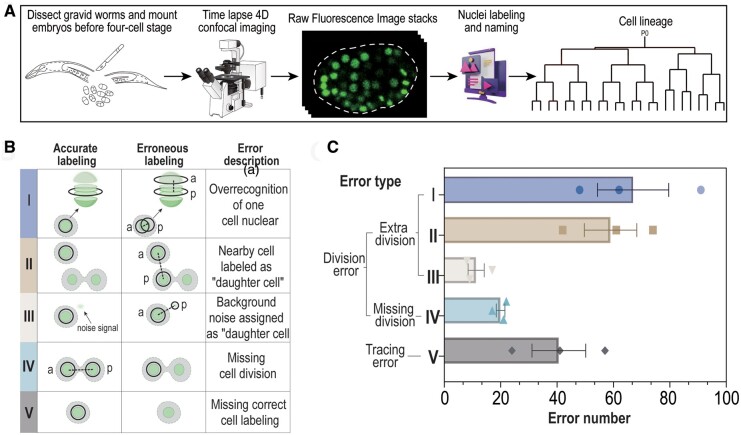
Characterization of errors by StarryNite in *C. elegans* embryo. (A) Pipeline of live cell imaging and automated cell lineage analysis. The automated nucleus tracing and naming is achieved by StarryNite with fluorescence-labeled nuclei as an input. Manual curation is needed to correct the errors in nucleus segmentation or tracing in order to build an accurate cell lineaging tree. (B) Nucleus labeling errors are categorized into five classes. Shown are the schematic representation of the comparisons between accurate and erroneous nucleus segmentation and tracing for each error class. Shown on the right is the description for each erroneous labeling. (C) Occurrence of each error class. The bar plots depict the mean number of each error class observed between approximately 160-cell to 360-cell stage during embryogenesis for three independent embryos.

To systemically understand the errors made by StarryNite during lineage tracing (Fig. 1A), we manually corrected the errors in three independent embryos which were imaged at 92 focal planes without pressure (Supplementary Table S1) and grouped them into broad categories, i.e. division error and tracing error (Fig. 1B). The former leads to either an extra or a missing division while the latter means StarryNite makes a false-negative call of nucleus that leads to the incorrect death of a cell. To gain an overview of different errors across various embryogenesis stages, we counted those between approximately 0- to 160-cell, 160- to 360-cell, and 360- to 550-cell stages (for ABalp lineage only) for three independent embryos. While most errors are division ones, the tracing errors, which are usually due to a poor SNR or very dim image, are also abundant (Fig. 1B and C and Supplementary Fig. S2). We further grouped the division errors into four classes, three of which led to extra division, while the last class caused missing division. Among the errors leading to the extra division, those usually with the highest occurrence were produced by over segmentation of nuclei, frequently on the *z* axis, which was referred to as class I (Fig. 1B and C, Supplementary Figs S2 and S3). The second highest class of error between approximately 0- to 160-cell and 160- to 360-cell stages, *i.e.* class II error, was mainly due to the movement of nuclei, leading to the proximity of two nonsister cells that were wrongly treated as a new sister pair, and calling of new division. The third class of error was caused by the false positive calling of a nucleus from background noise, which is the least observed errors out of all classes (Fig. 1B and C and Supplementary Figs S2 and S3). Both class IV and V errors were caused by a false negative call of a nucleus. The former results in a missing division whereas the latter leads to a false cell death, which is the third most common classes of errors (Fig. 1C and Supplementary Fig. S3). We did not characterize the “false diameter” type of error as Aydin *et al.* (2010) did because such errors barely influence the lineaging construction as long as the cell calling is accurate. Notably, the error rate likely varies depending on factors including differences in image quality, imaging strain, microscope, or StarryNite parameters; however, the types of errors should not deviate significantly.

### 3.2 Pipeline and image-wise evaluation of *DELICATE*

**Figure 2.**
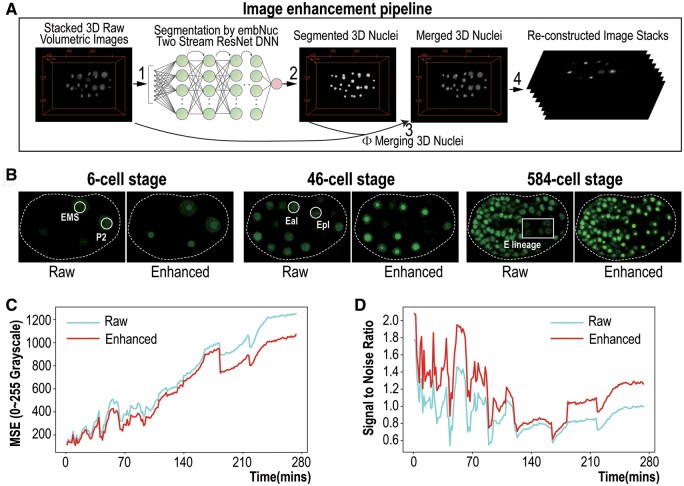
Image enhancement workflow of *DELICATE*—Deep learning cell nuclei enhancing technology. (A) Schematics showing the pipeline of *DELICATE*. 2D fluorescent image stacks are first constructed as raw 3D volumetric blocks. The blocks are used as the input for a fast and light deep learning model, which is customized for time-lapse 3D cell nuclei images, to generate segmented 3D nuclei blocks. The raw and segmented 3D image blocks are subsequently merged, formulated and re-sectioned into 2D image stacks that are used as input for StarryNite. (B) The comparison of nuclei signal intensities between raw and enhanced images across different embryonic developmental stages. Eggshells are indicated with white dashed lines. Note that E lineage cells before and after image enhancement are highlighted in white rectangular area. (C) Comparison of Mean Square Error (MSE) between raw and enhanced images for one embryo across time. Shown is the Mean Square Error (MSE) value of individual time point from 0 to 280 mins. Note that a lower MSE depicts less signal discrepancies from ground truth images. (D) Comparison of Noise Ratio (SNR) measurements of raw and enhanced images for the same embryo as in (C) across time. Note that a higher SNR means more signals and less noise for nuclei tracing.

To systematically reduce the errors generated from StarryNite during lineage tracing, we developed an automated pipeline to improve the image quality, *i.e.DELICATE*, and tested the utility of the improved images using the existing image segmentation algorithm, StarryNite, for automated tracing of cell lineage of *C. elegans* embryogenesis. The models, algorithms, and corresponding software in the pipeline were built with pre-trained deep neural network (DNN)-based image processing algorithm and fitted on raw fluorescence nuclei images of *C. elegans*. The dataflow and workflow were demonstrated in Fig. 2A and Supplementary Fig. S1. Step 1, the 2D raw images were stacked and reconstructed to the 3D volumetric images with a uniform imaging resolution (0.18 µm) through interpolation in *z* axis. Then the 3D images were subject to per-image intensity contrast refinement. Step 2, the 3D images were processed by the segmentor-based DNN (deep neural network, designed for segmentation), and the network produced the detection results for every single nucleus object (region), with clear boundary between each other (foreground and background). The detected/segmented nucleus is a connected sphere-like region, which is a pseudo-nucleus object created from the nucleus fluorescence signals. Step 3, the artificially generated (segmented, pseudo-nuclei) 3D images were sliced (cut with corresponding *z*-axis intervals) to 2D images, for which the signal intensity can be adjusted arbitrarily, usually the highest intensity of nuclei fluorescence from the slide in the current time point. Step 4, *DELICATE* combined the raw 2D fluorescence images, $I_{\mathrm{raw}}$, with the corresponding 2D pseudo-nuclei images, $I_{\mathrm{pseudo}}$, sliced from the pseudo-nuclei spheres with merging operation $\Phi \;\left(I_{\mathrm{raw}},I_{\mathrm{pseudo}}\right)$, for the final “enhanced” 2D fluorescence images $I_{\mathrm{enhanced}}$. With this combination, we kept the raw and real nuclei signals when the DNN fails to detect/segment A nuclei region (Fig. 2A). The enhanced 2D images maintain a complete correspondence with the original ones, but demonstrate a relatively constant SNR and brightness across cells, which facilitates StarryNite for cell lineage tracing. It takes approximately 60 min to complete the automated processing of an embryo with 300 time-point imaging up to around 550-cell stage. The testing computer run with Windows 10, on Intel i7-9700 CPU @ 3.00 GHz and 16 GiB physical memory.

For evaluating the enhanced images, we conducted both qualitative and quantitative comparison. The brightness of the most blurred sublineages D, E, and germline progenitors, P4, Z2, and Z3, has been remarkably enhanced (Fig. 2B and Supplementary Fig. S3), especially during the later stages when the cells are small in size but large in number, and densely positioned. We used two metrics for image-wise comparisons, MSE and SNR (see Sections 2 and 3). Compared with the raw images $I_{\mathrm{raw}}$, the enhanced images $I_{\mathrm{enhanced}}$ demonstrate a greatly reduced pixel-wise MSE and an increased image-wise SNR (Fig. 2D), throughout embryogenesis. Specifically, we observed a consistent smaller value in MSE over development relative to that before treatment with *DELICATE*, especially after approximately 170 min of imaging when the embryo develops from 350-cell stage to 550-cell stage, suggesting that *DELICATE* treatment would reduce errors mostly after 350-cell stage (Fig. 2C and Supplementary Fig. S4A). To further evaluate the benefit of *DELICATE* treatment on the cell lineage analysis, we compared SNR of the fluorescence images over time before and after processing with *DELICATE*. Again, we observed a consistent higher SNR over development relative to that before treatment with *DELICATE*, especially after approximately 170 min of imaging, suggesting that *DELICATE* treatment would greatly reduce error rate after 350-cell stage (Fig. 2D and Supplementary Fig. S4B).

To demonstrate the advantages of *DELICATE* over other commonly used image enhancement tools, we conducted a qualitative comparison between *DELICATE* and two widely adopted tools, i.e. Fiji (ImageJ) and Huygens (a deconvolution tool integrated in Leica software). We found that *DELICATE* exhibited specific improvements relative to the other two tools, which may facilitate more accurate cell recognition from the StarryNite software (Supplementary Fig. S5). Specifically, while the other tools only modified the global image contrast, *DELICATE* allowed for the highlighting of the central position of every single cell nucleus (Supplementary Fig. S5), thereby reducing overlapping fluorescence signals and removing ambiguous signals from cell-cell contacts, particularly for samples with a high ratio of nuclei size to cell size.

### 3.3 *DELICATE* reduces all classes of errors made by StarryNite

**Figure 3.**
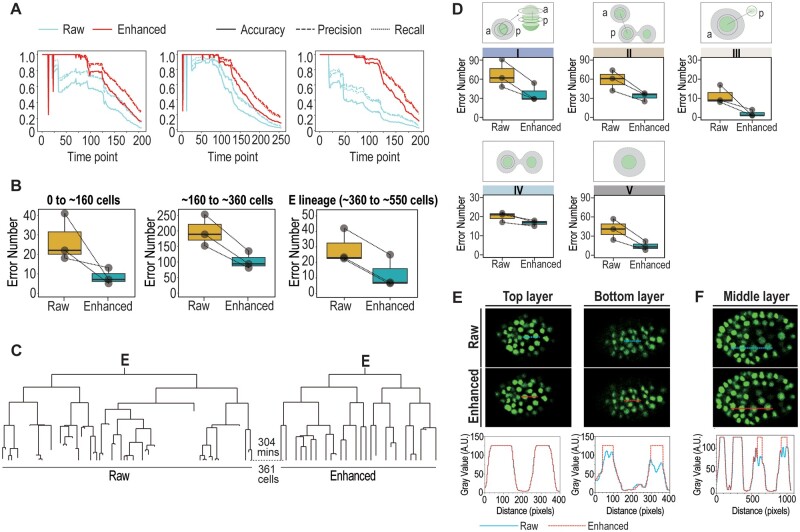
A decrease in the occurrence of errors in nuclei segmentation and tracing after image enhancement. (A) Comparison of accuracy, precision and recall between raw and enhanced images for three independent embryos across time. (B) Quantification of errors before and after image enhancement across three consecutive embryogenesis intervals. The paired box plots show the mean number of total errors before and after image enhancement for three embryonic stages: i.e. 0 to ∼160 (left), ∼160 to ∼360 (middle), and ∼360 to ∼550 cells (right). Shown are the data from the embryos shown in (A). Error numbers for the same embryo before and after image enhancement are connected by dashed line. Note that only the error numbers from the E lineage are counted in the ∼360 to ∼550 cells stage for simplicity. (C) Comparison of E sub-lineage trees output by StarryNite without manual curation before (left) and after image enhancement (right). (D) Nearly all classes of errors are substantially reduced after image enhancement. The paired box plots show the mean number of errors before and after imaging enhancement counted from ∼160 to ∼360-cell stages for three independent embryos. Error numbers for the same embryo before and after image enhancement are connected by dashed line.

To systematically quantify the effect of *DELICATE* on the reduction of errors made by StarryNite, we first calculated the accuracy, precision and recall (see Sections 2.3) for images of the same three embryos as those used for the error characterization with *DELICATE* before and after image enhancement. All the three values were remarkably elevated across each time point for all the three embryos (Fig. 3A, Supplementary Table S3), indicating an overall improvement of cell-tracing accuracy after enhancement. We next comprehensively counted and contracted the total number of errors made by StarryNite from early (0- to 160-cell), mid (160- to 360-cell), and late embryogenesis (360–550 cell, E and ABalp lineage) in the three embryos before and after *DELCIATE* treatment (Fig. 3B and Supplementary Fig. S6). Consistently, we observed a remarkably reduced number of errors by nearly a half across all developmental stages, which is evident from the raw lineage tree output by StarryNite without any curation (Fig. 3C, Supplementary Figs S7–S9). Moreover, the lineage-wise illustrations provide a qualitative and intuitive comparison of lineage tracing accuracy between raw images and enhanced images (Supplementary Figs S7 and S8).

To investigate whether there is any bias in terms of error reduction between different classes of errors, we compared the numbers for each class of errors in the three embryos from 0- to 160-cell and 160- to 360-cell stages based on the fluorescence nuclei images. We found that most classes of errors show a reduction of approximately by a half except for the class IV, which shows only about 10% reduction in error number (Fig. 3D and Supplementary Fig. S9). To further understand how the images processed by *DELICATE* show a better performance in image segmentation, we performed direct measurement of gray value across adjacent nuclei. We found that inherent fluorescent intensity heterogeneity across cells in each time point and focal plane can be greatly improved. For example, the dim signals at the bottom layer of the sample slide, caused by more severe light scattering during confocal microscopy, were increased substantially and comparable to those at the top layer with no background disturbance (Fig. 3E). Similarly, the dim expression of the florescent marker in the E cells from the middle layer can be enhanced to achieve a uniform intensity compared with neighboring cells (Fig. 3F).

Finally, to evaluate whether *DELICATE* enables broad applications, we applied the method to *C. elegans* embryos labeled with mCherry markers (Ho *et al.* 2015) and imaged using a spinning disc confocal microscope (Ma *et al.* 2021). These data were retrieved from previously published data of our own and others. We demonstrated remarkable enhancement of nuclei signal and greatly improved cell tracing performance from StarryNite for both datasets (Supplementary Figs S10 and S11) using *DELICATE*, indicating the versatility of our framework across various cell lineaging platforms. Taken together, we devised a framework, *DELICATE*, for the enhancement of nucleus fluorescence images in *C. elegans* embryo, which considerably reduces the number of various types of errors made by StarryNite. Therefore, it reduces the time for manual correction by at least a half to perform automated cell lineage analysis. The framework is expected to improve the segmentation performance of other types of images that demand a high SNR.

## 4 Discussion

Automated cell lineage tracing with time-lapse 3D fluorescence images of a live organism emerges as an essential tool for in-depth study of regulatory control of developmental process with cellular resolution at 1-*min* interval. Existing algorithms for nucleus segmentation and tracing often suffer from a higher error rate, which demands substantial human interventions to manually correct the errors before a decent cell lineage can be achieved. These errors are mainly due to the crowdedness of the labeled nuclei that are small and undergo rapid movement, which makes it difficult to accurately distinguish a real division from a cell movement. In addition, a relatively poor image quality imposed by a tradeoff between embryo viability and photobleaching and/or phototoxicity also negatively impacts the accurate segmentation. Furthermore, if nuclei are sampled in a higher *z* resolution, e.g. for the reconstruction of cellular shape (Cao *et al.* 2020), even more time is needed for curation up to 550-cell stage. On top of this, the uneven fluorescence intensity and the size of nuclei across cell types and developmental stages further complicate the effective segmentation of nuclei. Most existing segmentation algorithms assume all the labeled nuclei as equal-sized with uniform labeling at least at the same developmental stage (Murray *et al.* 2006), which leads to a substantial amount of errors.

Deep learning (DL) is a promising technology for enhancing customized images across various fields (Cao *et al.* 2020, Duanmu *et al.* 2022, Malin-Mayor *et al.* 2022, Yaacoub *et al.* 2022, Meirelles *et al.* 2023). Here we apply DL in improving cell lineage tracing in *C. elegans* embryo, in which we leverage deep spatial features and prior knowledge about the spatial and temporal arrangement of the fluorescence signals through DL to artificially enhance the SNR and local intensity of nuclear fluorescence signal. This results in a remarkably improved quality of the images, which dramatically increases the accuracy of cell tracing by StarryNite. Although various efforts have been made to improve the software tools used in cell lineaging tracing pipeline (Boyle *et al.* 2006, Santella *et al.* 2010, 2014, Katzman *et al.* 2018), none have focused on the initial enhancement of raw embryogenesis images. Our DL-based *DELICATE* framework addresses this gap and holds promise for enabling more accurate lineage when used in conjunction with previous algorithmic advancements. Our framework also significantly reduce the error rate by any other segmentation algorithms that process time-lapse images with fast movement and those that demand a high SNR of images and even distribution of signal intensity and object size. It is worth noting that we may not have fully optimized the StarryNite parameters for nonpressurized embryos, which may have partially contributed to the elevated error rate. Further optimization of these parameters may help further reduce the error rate.

Moreover, fluorescent light attenuation is a common issue, especially in long-term multi-layer imaging, which can be due to heterogenous fluorescent expression in different cells (Harikumar *et al.* 2017), photobleaching of fluorescent proteins (Hirano *et al.* 2022) or light scattering from thick samples (Stanciu *et al.* 2010). Traditional methods such as the development of more photostable fluorophores (Hirano *et al.* 2022) and developing software-based light compensation methods (Čapek *et al.* 2006) have various limitations. Here, we establish a DL-based image enhancement technique, which can facilitate qualitative comparisons of diverse cellular activities indicated by fluorescence signals, without the need for extensive biological optimization or costly software upgrades.

In conclusion, we establish an effective pre-trained DL method *DELICATE* that efficiently improves the accuracy of automated cell lineage tracing by StarryNite, which could be applicable to any other segmentation algorithm with limited modification. Consequently, the method greatly reduces the efforts for manual correction of errors produced by StarryNite, especially at the late *C. elegans* embryo up to the 550-cell stage, enabling a fast and accurate generation of cell lineages at large scale.

## Supplementary Material

btae626_Supplementary_Data

## Data Availability

The DELICATE code and user manual were deposit in GitHub: https://github.com/plcx/NucApp-develop. A demonstration of the user interface and the sample image for training using customized image are shown in Supplementary Fig. S12. The training and evaluation data were available on https://doi.org/10.6084/m9.figshare.26778475.v1. The other data underlying this will be shared on reasonable request to the corresponding author.
