# Supplementary material for: Deep learning-based enhancement of fluorescence labeling for accurate cell lineage tracing during embryogenesis
Source: Bioinformatics. 2024 Oct 17;40(11):btae626. doi: 10.1093/bioinformatics/btae626 (PMC11549013; doi:10.1093/bioinformatics/btae626)
Supplement: btae626_Supplementary_Data [file btae626_supplementary_data.zip › Suppl_Zhao_bioinformatics_MS_accepted.pdf]

Supplementary Materials of

Deep Learning-based Enhancement of Fluorescence Labeling for Accurate Cell Lineage Tracing During Embryogenesis

Zelin Li[1,2†], Dongying Xie[3†], Yiming Ma[3†], Cunmin Zhao[3], Sicheng You[2],

Hong Yan[1,2], Zhongying Zhao[3*]

1. *Department of Electrical Engineering, City University of Hong Kong, Hong Kong, China*
2. *Centre for Intelligent Multidimensional Data Analysis, Hong Kong Science Park, Hong Kong, China*
3. *Department of Biology, Hong Kong Baptist University, Hong Kong, China*

† *These authors contribute to the manuscript equally.*

* *Corresponding authors. E-mail: zyzhao@hkbu.edu.hk*

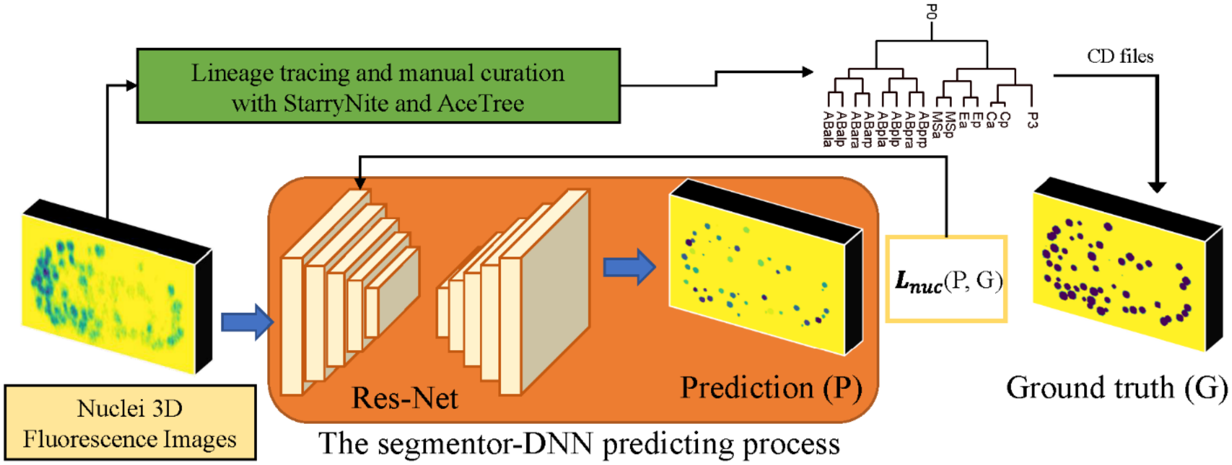

Figure S1. Schematic of the workflow of DELICATE.

The ground truth generation, training, and image enhancement running processes are demonstrated. The $L_{nuc}(P, G)$ is the loss function and training target for the DNN

network. Prediction is the results generated by the DNN by learning from the Ground truth. CD files are the lineage tracing for contract our Ground truth.

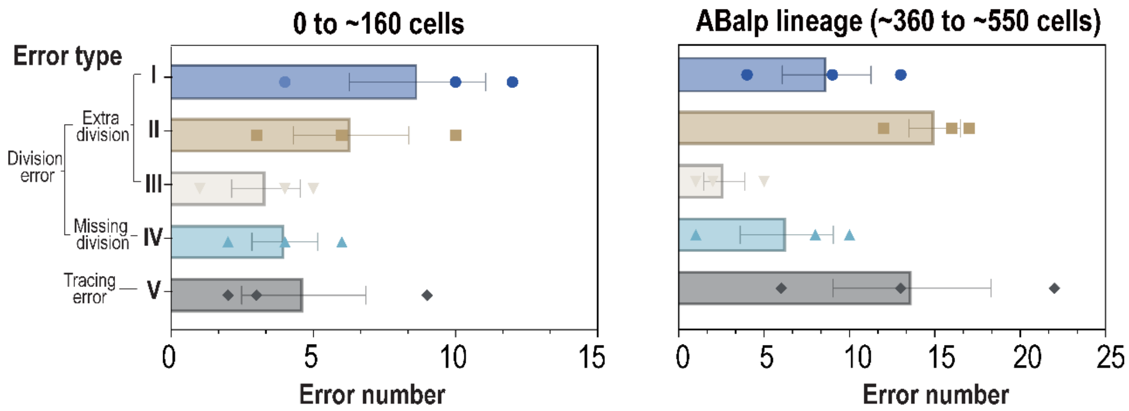

Figure S2. Occurrence of each nucleus labelling error.

The bar plots depict the mean number of each error class observed between approximately 0 to 160-cell stage (left) and between approximately 360 to 550-cell stage in ABalp cell lineage (right) during embryogenesis for three independent embryos.

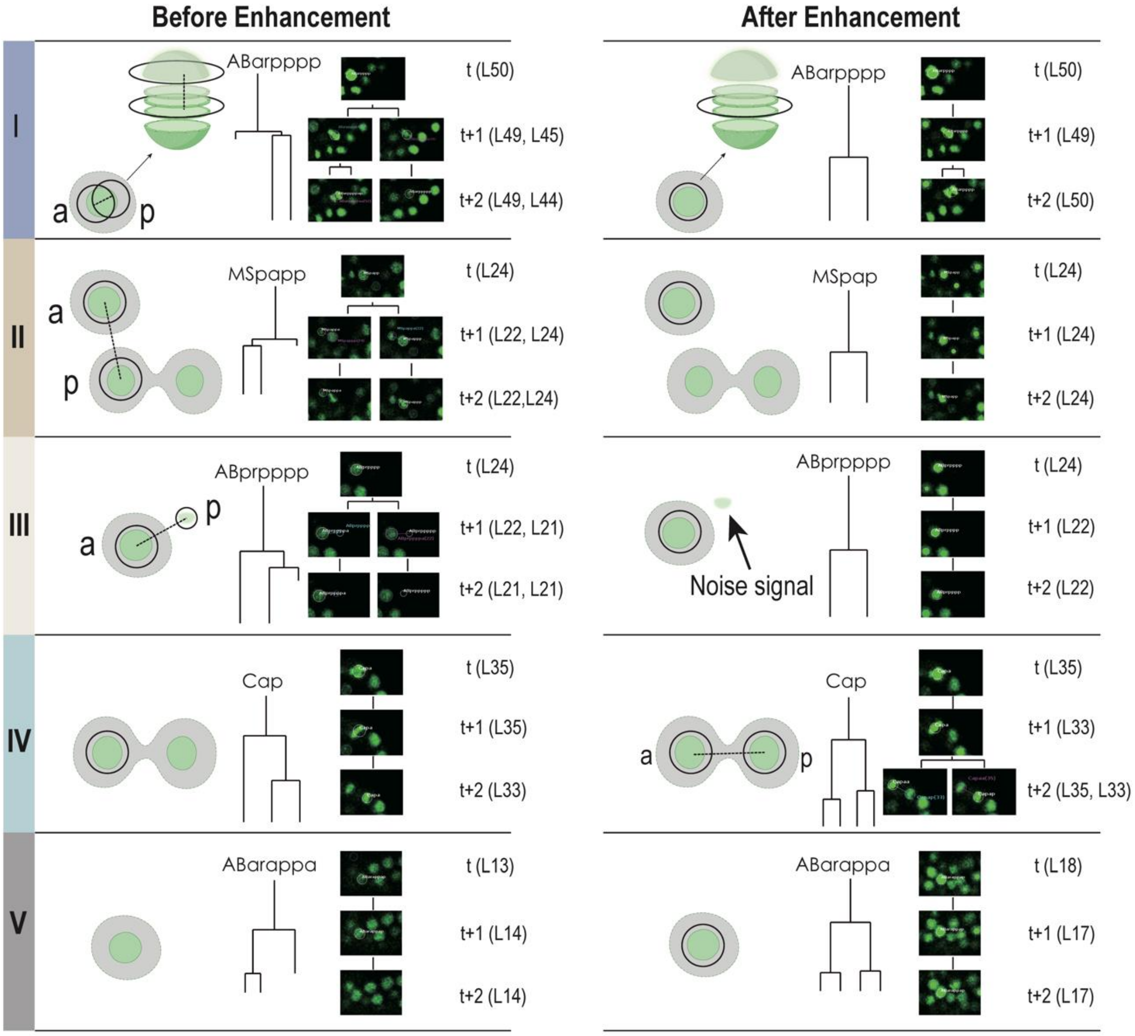

Figure S3. Raw fluorescence images associated with errors shown in Fig. 1B.

For each class of error, one typical sublineage is shown before (left) and after nuclei image enhancement (right). The cell nuclei are automatically recognized and named by StarryNite and AceTree.

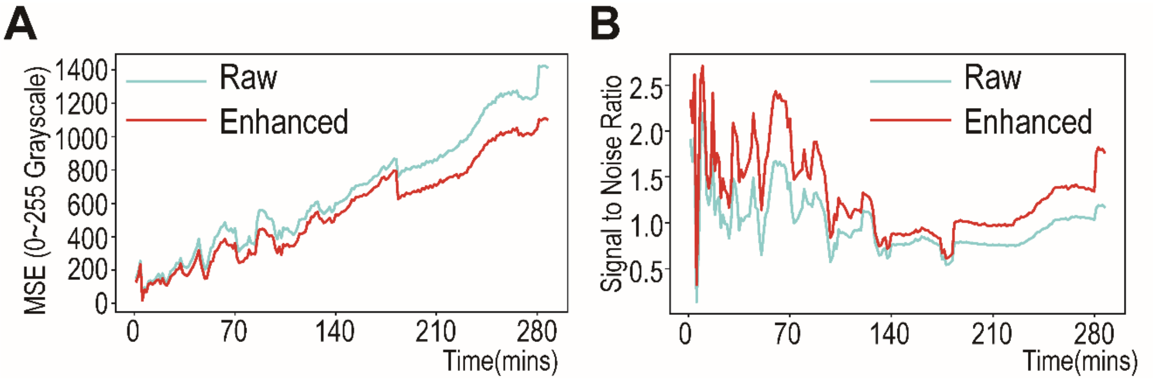

Figure S4. Comparable enhancement of image quality for another independent embryo as those shown in Fig. 2B and C.

(A) Comparison of Mean Square Error (MSE) between raw (cyan) and enhanced images (red) for the embryo across time. Shown is the Mean Square Error (MSE) value of individual time point from 0 to 280 minutes.

(B) Comparison of Noise Ratio (SNR) measurements of raw (cyan) and enhanced images (red) for the same embryo as in (A) across time.

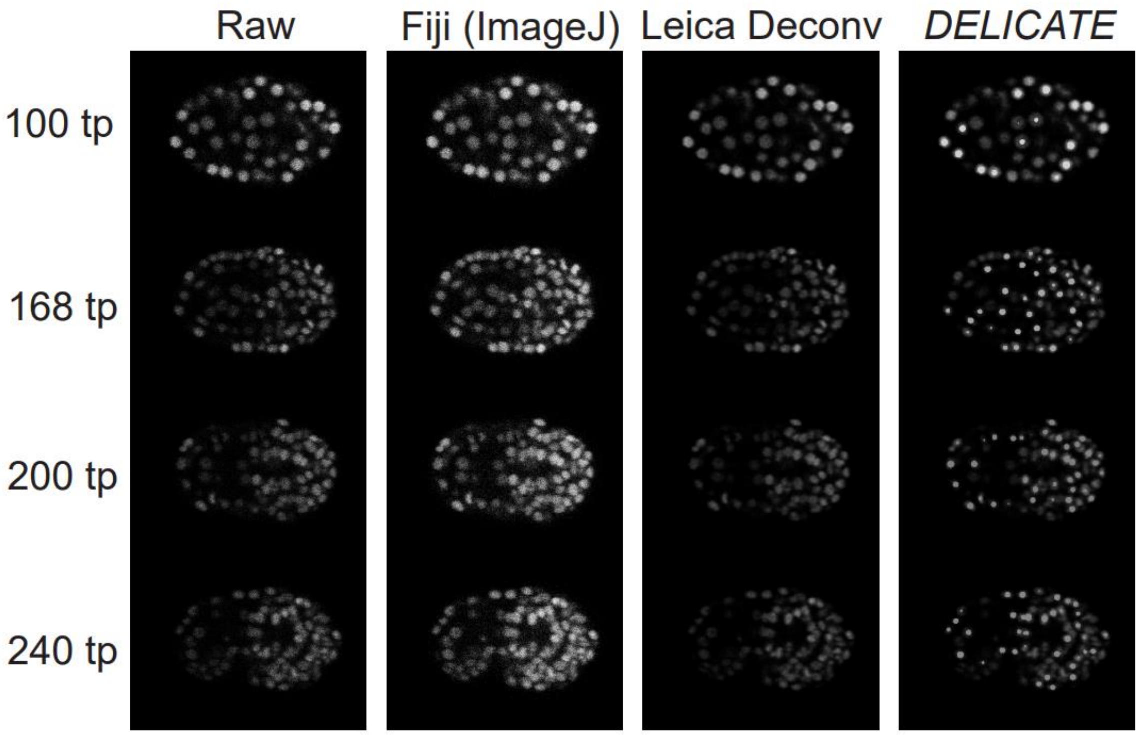

Figure S5. Comparison between *DELICATE* and other image enhancement methods.
Shown are the time-lapse raw images and the corresponding images enhanced by Fiji

(ImageJ), Leica deconvolution software (Huygens) and *DELICATE* across different time point. Tp: time point.

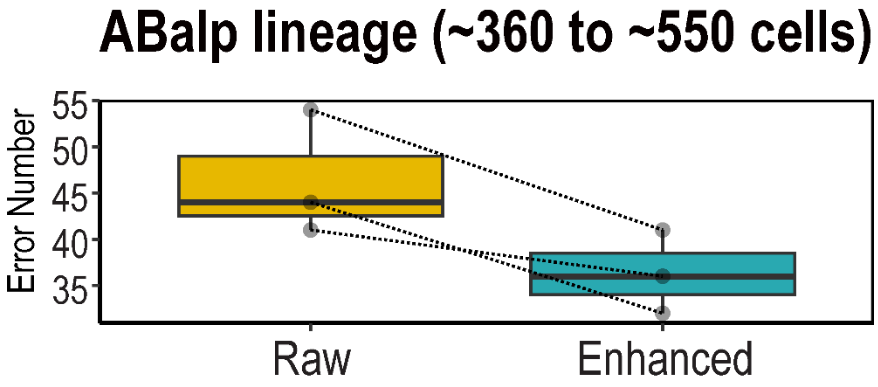

Figure S6. Quantification of total errors before (brown) and after image enhancement (green) in ABalp lineage for ~360-~550 cell-stage.

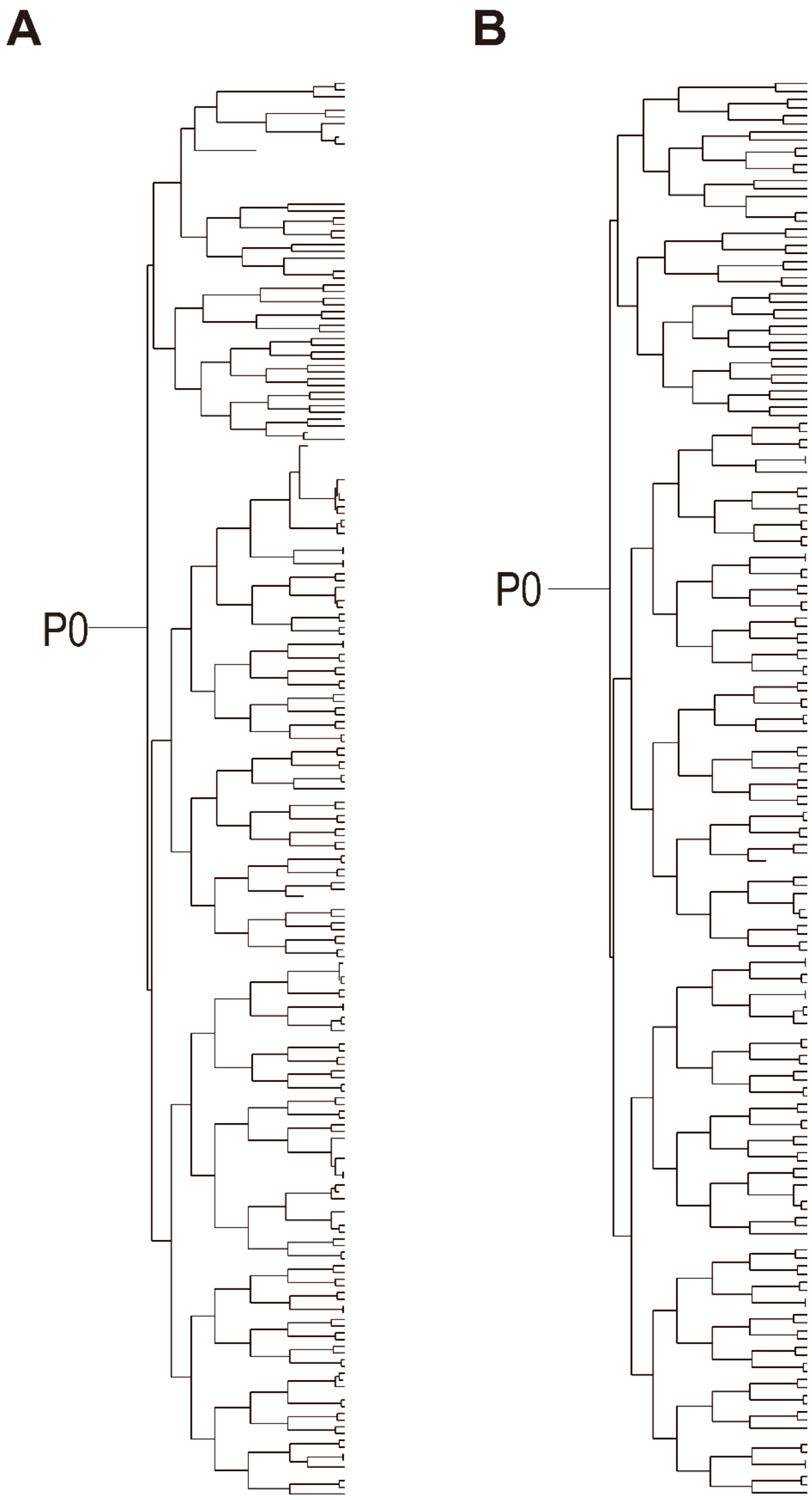

Figure S7. Comparison of lineage tree up to ~160-cell stage from the same embryo output by StarryNite without manual curation before (A) and after image enhancement (B).

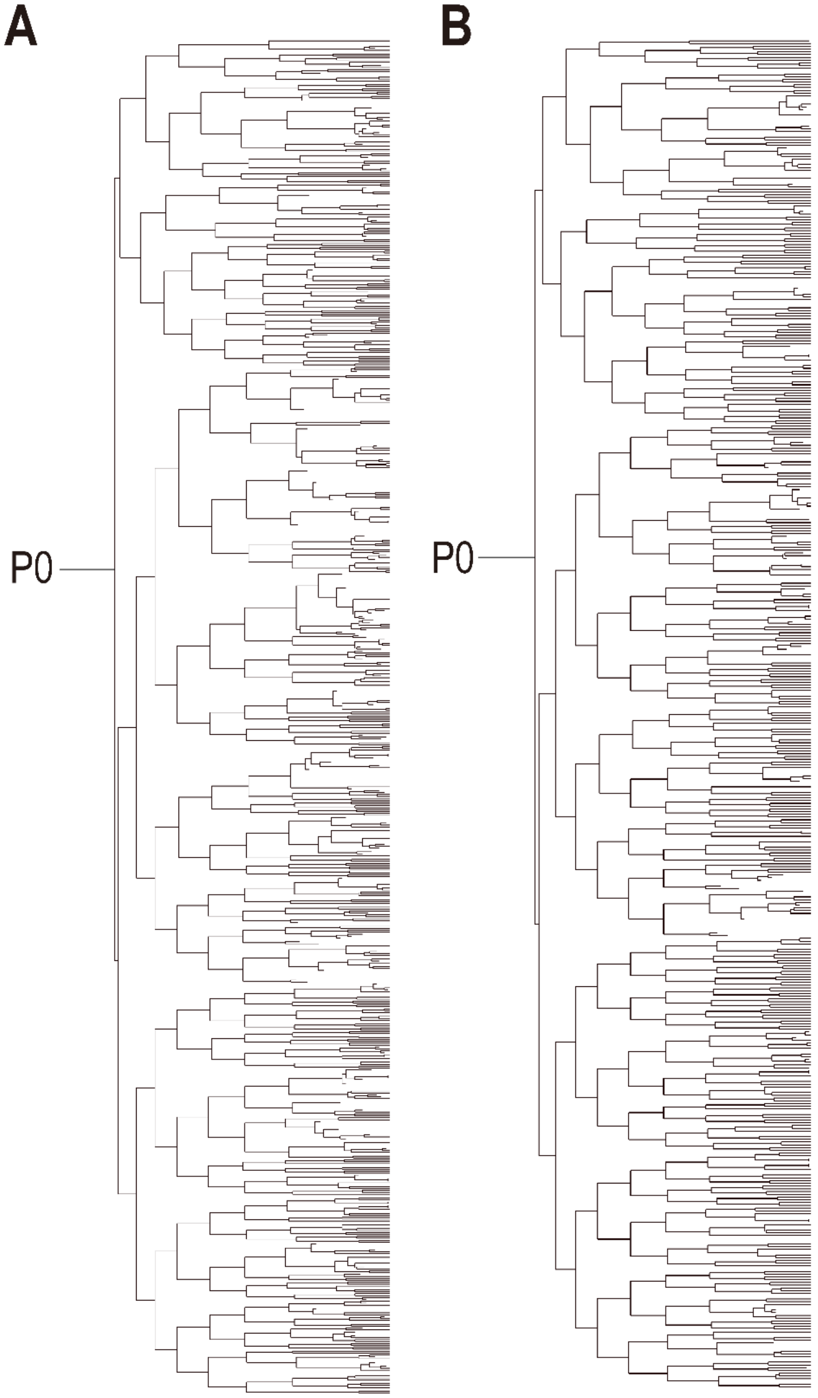

Figure S8. Comparison of lineage tree from ~160 to ~360-cell stage of the same embryo output by StarryNite without manual curation before (A) and after image enhancement (B). Note that the all the errors from ~4 to ~160-cell stage were manually curated.

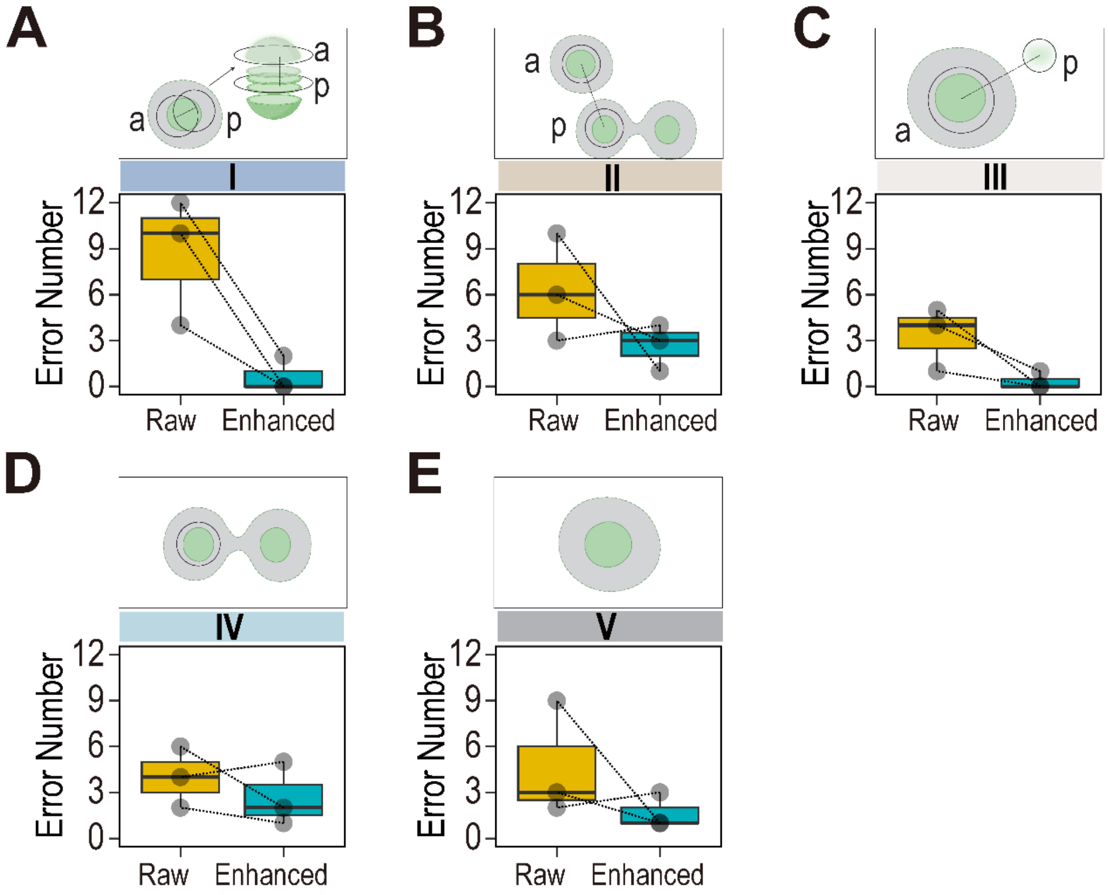

Figure S9. Nearly all classes of errors are substantially reduced after image enhancement as in Figure 3B except that errors were counted from ~4 to ~160-cell stages.

The paired box plots show the mean number of errors before (brown) and after imaging enhancement (green) for three independent embryos each. Error numbers for the same embryo before and after image enhancement are connected by dashed line.

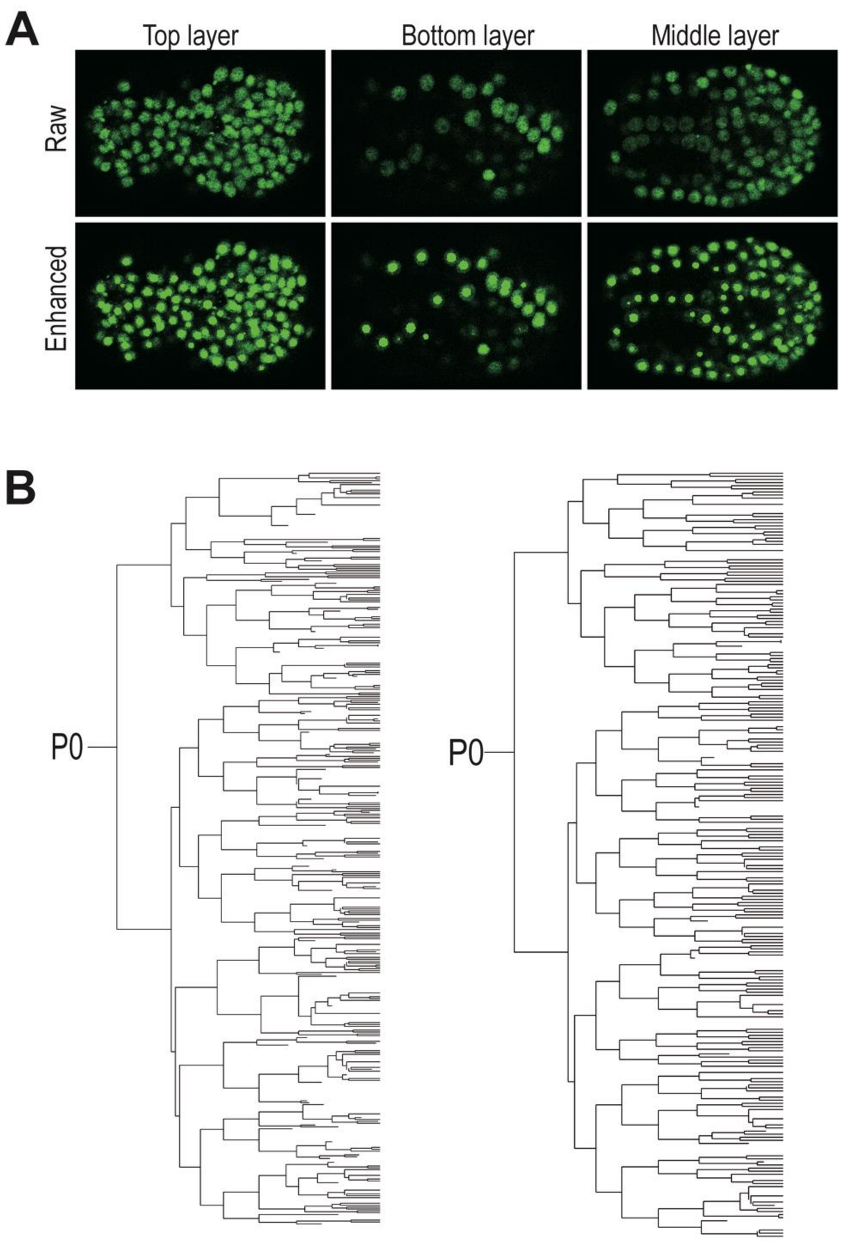

Figure S10. DELICATE allows image enhancement of embryos labeled with different fluorescent-marker.

(A) Representative images for the comparison of nuclei signal intensities between raw and enhanced images for top layer (left), middle layer (middle) or bottom layer (bottom) of an *C. elegans* embryo labeled with mCherry reporter (pseudo-colored as green).

(B) Comparison of the lineage tree output by StarryNite for the same embryo shown in (A) from 0 to ~200-cell stage before (left) and after image enhancement (right). Note that no manual curation was performed.

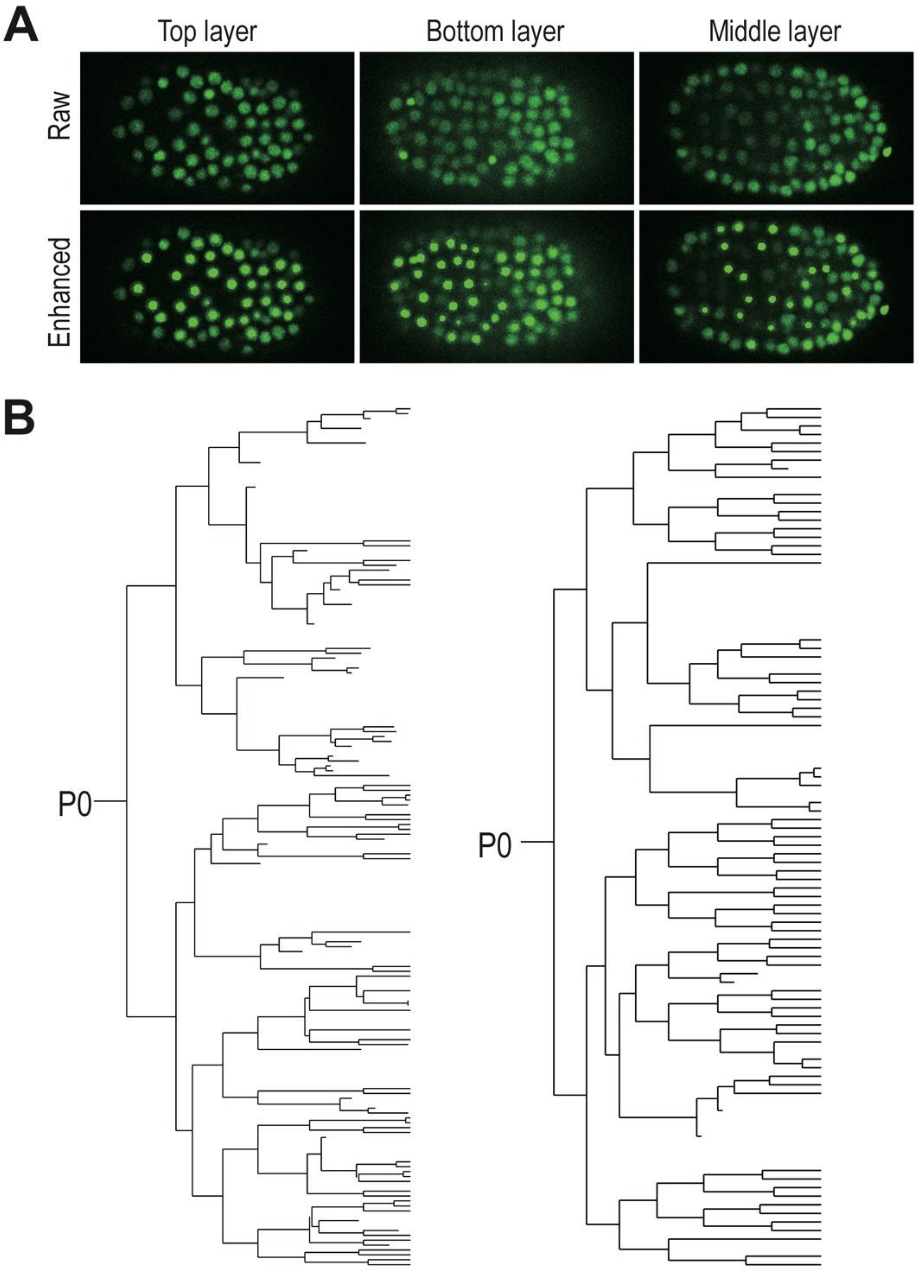

Figure S11. DELICATE allows image enhancement of embryos imaged with different microscope.

(A) Representative images for the comparison of nuclei signal intensities between raw and enhanced images for top layer (left), middle layer (middle) or bottom layer (bottom) of an *C. elegans* embryo imaged using the spinning disc confocal microscope.

(B) Comparison of the lineage tree output by StarryNite for the same embryo shown in (A) from 0 to ~100-cell stage before (left) and after image enhancement (right). Note that no manual curation was performed.

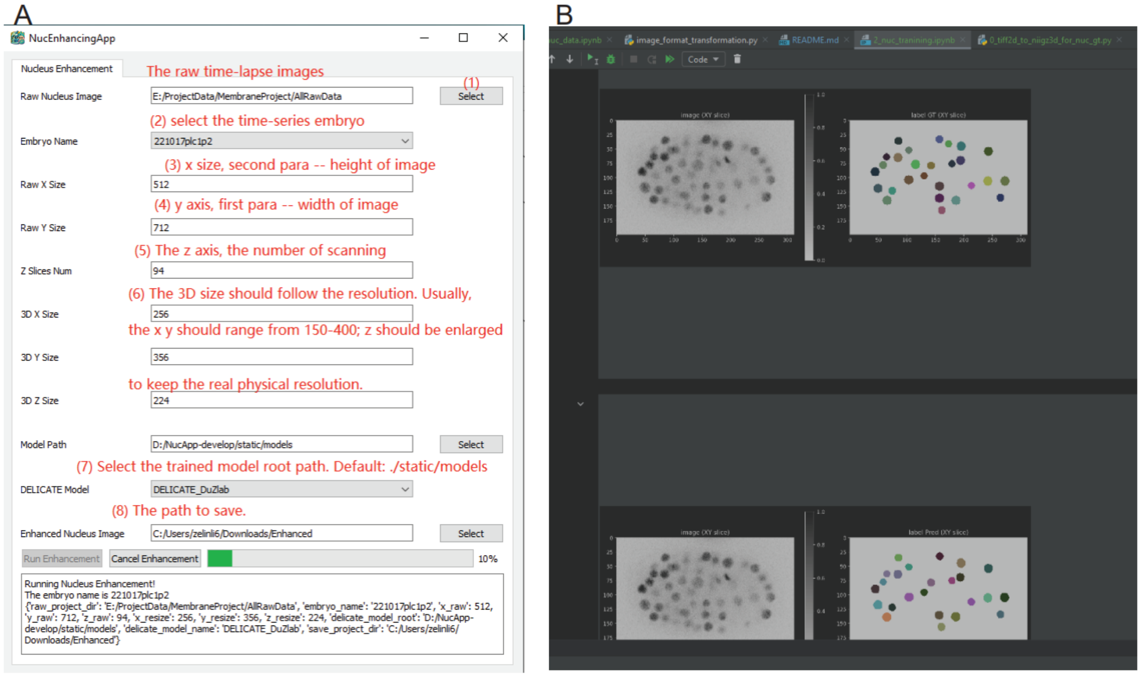

Figure S12. Demonstration of *DELICATE* software user interface and data training process.

(**A**) Detailed steps for running *DELICATE* using user interface.

(**B**) Validation of visualization results after the training process for pretrained DELICATE_DuZLab model.
