# Supplementary material for: Deep learning-based enhancement of fluorescence labeling for accurate cell lineage tracing during embryogenesis
Source: Bioinformatics. 2024 Oct 17;40(11):btae626. doi: 10.1093/bioinformatics/btae626 (PMC11549013; doi:10.1093/bioinformatics/btae626)
Supplement: btae626_Supplementary_Data [file btae626_supplementary_data.zip › Table S2 StarryNite parameters.pdf]

```
%parameter file for matlab nuclear detection
conservememory=false;
slices=92;
xyres=0.09;
zres=0.43;
start_time=1;
end_time=240;
firsttimestepdiam=80;
firsttimestepnumcells=4;
parameters.staging=[50,120,280,300,350];
parameters.intensitythreshold=[0.004,0.005,0.008,0.001,0.008,0.008];
%RS_2022newconfocal_zelin_parameters.intensitythreshold=[0.02,0.015,
0.04,0.039,0.039,0.04];
parameters.sigma=1;
parameters.rangethreshold=[84,31,21,20,10,8];
parameters.nndist_merge=[.8,.65,.6,.63,.5,.4];
parameters.mergelower=[-200,-100,-75,-95,-90,-80];
parameters.split=[15,9,6,5,3,2];
parameters.armerge=[1.6,1.6,1.3,1.2, 1.2,1.0];
parameters.mergesplit=[1,.5,.4,.4,.5,.6];
parameters.large_ray_threshold=1.5;
parameters.small_ray_threshold=1/3;
parameters.boundary_percent=.2;
ROI=false;
ROIxmin=0;
ROIxmax=0;
ROIymin=0;
ROIymax=0;
newscope=false;
distribution_file='clean_distributions.mat';
distribution_file2='clean_distributions.mat';
LSM=false;
outputSlice=false;
parameters.GRADIENT_THRESH=false;
nodata=true;
nodatause=true;
savedata=true;
singlevolume=false;
SNoutput=true;
downsampling=0.5;
```
